# Localised release of matrix metallopeptidase 8 in fatal cerebral malaria

**DOI:** 10.1002/cti2.1263

**Published:** 2021-04-29

**Authors:** Athina Georgiadou, Praveena Naidu, Sophie Walsh, Steve Kamiza, Valentina Barrera, Simon P Harding, Christopher A Moxon, Aubrey J Cunnington

**Affiliations:** ^1^ Department of Infectious Disease Section of Paediatric Infectious Disease Imperial College London London UK; ^2^ Centre for Paediatrics and Child Health Imperial College London London UK; ^3^ Department of Pathology College of Medicine University of Malawi Blantyre Malawi; ^4^ Department of Eye and Vision Science Institute of Life Course and Medical Sciences Liverpool University Hospitals Foundation Trust Members of Liverpool Health Partners University of Liverpool Liverpool UK; ^5^ Wellcome Centre for Integrative Parasitology Institute of Infection, Immunity and Inflammation University of Glasgow Glasgow UK; ^6^ Department of Paediatrics College of Medicine University of Malawi Blantyre Malawi; ^7^ Malawi‐Liverpool Wellcome Clinical Research Programme College of Medicine University of Malawi Blantyre Malawi

**Keywords:** cerebral malaria, matrix metalloproteinase 8, neutrophils, pathogenesis

## Abstract

**Objective:**

Cerebral malaria (CM) is a complication of *Plasmodium falciparum* malaria, in which progressive brain swelling is associated with sequestration of parasites and impaired barrier function of the cerebral microvascular endothelium. To test the hypothesis that localised release of matrix metallopeptidase 8 (MMP8) within the retina is implicated in microvascular leak in CM, we examined its expression and association with extravascular fibrinogen leak in a case–control study of post‐mortem retinal samples from 13 Malawian children who met the clinical case definition of CM during life. Cases were seven children who were found on post‐mortem examination to have ‘true‐CM’ (parasite sequestration in brain blood vessels), whilst controls were six children who had alternative causes of death (‘faux‐CM’, no parasite sequestration in blood vessels).

**Methods:**

We used immunofluorescence microscopy and independent scoring, by two assessors blinded to the CM status, to assess MMP8 expression, extravascular fibrinogen as an indicator of vascular leak and their co‐localisation in the retinal microvasculature.

**Results:**

In ‘true‐CM’ subjects, MMP8 staining was invariably associated with sequestered parasites and a median of 88% (IQR = 74–91%) of capillaries showed MMP8 staining, compared with 14% (IQR = 3.8–24%) in ‘faux‐CM’ (*P*‐value = 0.001). 41% (IQR = 28–49%) of capillaries in ‘true‐CM’ subjects showed co‐localisation of extravascular fibrinogen leak and MMP8 staining, compared with 1.8% of capillaries in ‘faux‐CM’ (IQR = 0–3.9%, *P*‐value = 0.01). Vascular leak was rare in the absence of MMP8 staining.

**Conclusion:**

Matrix metallopeptidase 8 was extensively expressed in retinal capillaries of Malawian children with malarial retinopathy and strongly associated with vascular leak. Our findings implicate MMP8 as a cause of the vascular endothelial barrier disruption in CM, which may precipitate fatal brain swelling.

## Graphical abstract

To test the hypothesis that localised release of matrix metallopeptidase 8 (MMP8) within the retina is implicated in microvascular leak in cerebral malaria (CM), we examined its expression and association with extravascular fibrinogen leak in a case‐control study of post‐mortem retinal samples from 13 Malawian children. MMP8 was extensively expressed in retinal capillaries of children with CM retinopathy and strongly associated with vascular leak. Our findings implicate MMP8 in the vascular endothelial barrier disruption in CM, which may precipitate fatal brain swelling.

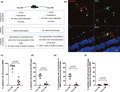

## Introduction

Cerebral malaria (CM) is characterised by coma and the detection of asexual blood‐stage malaria parasites, without other identifiable cause.^1^ In fatal cases, the pathognomonic histological feature is sequestration of parasitised erythrocytes in the microvasculature of the brain and retina, the latter being strongly associated with a characteristic malarial retinopathy which can be visualised during life.^1^ Recent studies have revealed that brain swelling is the final common pathway to death in paediatric CM, occurring in close association with rapid fluid egress from focal leaks in the blood–retinal barrier and with histological evidence of similar microvascular leak in the post‐mortem brain.^1^, ^2^, ^3^, ^4^ The molecular mechanisms which disrupt vascular integrity in human CM are not well established but may be important therapeutic targets.

Recent studies have implicated the expression of neutrophil granule protein genes, particularly matrix metalloproteinase 8 (*MMP8*), in the pathogenesis of CM.^5^, ^6^, ^7^ MMP8 is one of the most abundant proteins in neutrophil tertiary granules, with collagenase activity, which can disrupt intercellular tight junctions and degrade the subendothelial basal lamina.^8^, ^9^ Previous work has shown that MMP8 expression can lead to breakdown of the blood–CSF barrier.^10^ Although MMP8 gene expression is increased in CM,^11^ and MMP8 is a plausible mediator of the vascular endothelial barrier disruption occurring in CM, systemic levels of MMP8 are similarly elevated in subjects with severe and uncomplicated malaria compared to healthy controls.^5^, ^7^ We have shown that mature blood‐stage malaria parasites, such as those sequestered in large numbers in the brain microvasculature in CM, can trigger release of MMP8 from neutrophils.^7^ We hypothesised that these sequestered parasites might stimulate localised release of MMP8 in the cerebral microvasculature, promoting localised vascular leak in CM, without necessarily increasing systemic MMP8 concentrations. In order to establish plausibility of MMP8 release as a cause of fatal brain swelling, we reasoned that there should be additional evidence that any vascular leak associated with MMP8 was initiated before death.

In order to test our hypothesis, we used retinal specimens from a unique collection of post‐mortem tissue from Malawian children who died from malaria and other causes. The pathognomonic histological features of CM are highly concordant between brain and retinal microvasculature in children with CM,^1^, ^4^, ^12^, ^13^ but only the retinal vasculature can be visualised in life to confirm the ante‐mortem origin of these features precedes fatal brain swelling. Therefore, we compared the association between MMP8 and leakage in the retinal microvasculature of children with retinopathy and histologically confirmed‐CM (‘true‐CM’) with children who were suspected to have CM at presentation but did not have retinopathy and who had an alternative cause of death reported at post‐mortem (‘faux‐CM’).

## Results

**Figure 1:**
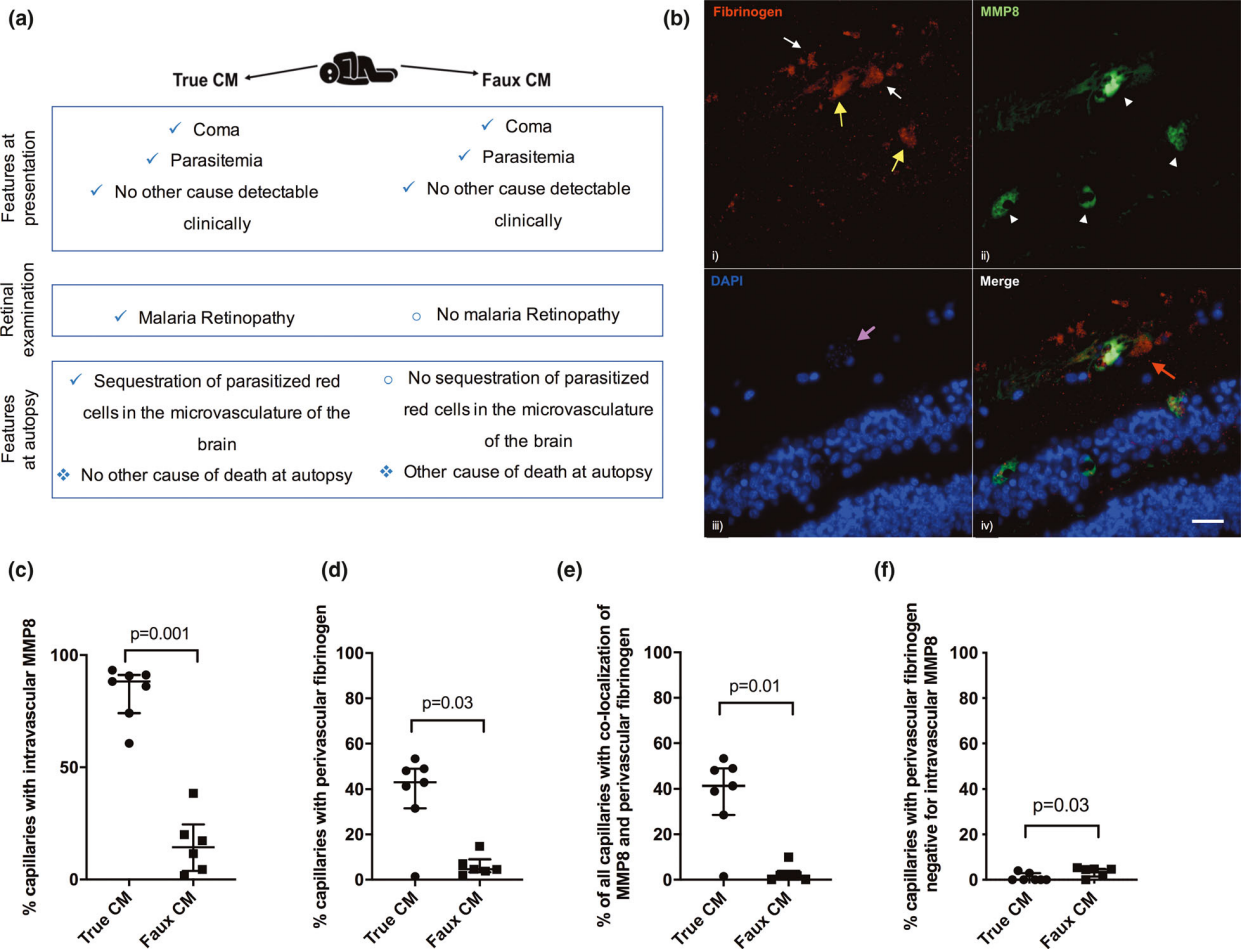
Matrix metallopeptidase 8 (MMP8) co‐localises with fibrinogen leak in the retinal capillaries in ‘true cerebral malaria’. **(a)** Illustration of the similarities and differences between ‘true’‐ and ‘faux’‐CM subjects. **(b)** Representative image showing co‐localisation of intravascular MMP8 and perivascular fibrinogen staining in the retina of a subject with true cerebral malaria (CM). (i) Fibrinogen (red) staining was seen intravascularly (yellow arrows), likely in areas of microthrombus formation, and immediately outside of capillaries (white arrows), indicating vascular leak. (ii) MMP8 (green) staining was observed intravascularly (arrowheads). (iii) DAPI (blue) stains nuclei, magenta arrow points to a capillary with heavy sequestration of parasitised erythrocytes (parasite nuclei visible as fine blue dots). (iv) Merged image shows co‐localisation of intravascular MMP8 and fibrinogen leak (red arrow) around one of the capillaries. Scale bar: 25 µm. **(c–f)** Quantification of intravascular MMP8 and perivascular fibrinogen staining in the retina of subjects with ‘true’‐ and ‘faux’‐CM. **(c)** Percentage of observed capillaries with intravascular MMP8 staining. **(d)** Percentage of observed capillaries with perivascular fibrinogen staining. **(e)** Percentage of all capillaries in each sample, with co‐localisation of intravascular MMP8 and perivascular fibrinogen. **(f)** Percentage of capillaries with perivascular fibrinogen staining which are negative for intravascular MMP8. Bars show median with interquartile range. Statistical analysis using the two‐sided Mann–Whitney test, *n* = 13; ‘True‐CM’, *n* = 7; ‘Faux‐CM’, *n* = 6.

All seven ‘true‐CM’ cases had characteristic retinopathy prior to death, whereas none of the six ‘faux‐CM’ controls had these retinal features (Table 1). Subjects with ‘true‐CM’ had a median age of 72 months (IQR = 25–89 months), while subjects with ‘faux‐CM’ had a median age of 24.5 months (IQR = 12.5–34.3 months; *P*‐value = 0.051). All subjects had positive peripheral blood smears for *Plasmodium falciparum* on admission. ‘True‐CM’ subjects had a median of 48 160 parasites per μL (IQR = 1729–308 720 parasites per μL) and ‘faux‐CM’ subjects had a median of 97 217 parasites per μL (IQR = 3301–430 230 parasites per μL; *P*‐value = 0.83). Five subjects with ‘faux‐CM’ had evidence of severe bacterial co‐infection at post‐mortem examination. Time from admission to death for ‘true‐CM’ subjects was a median of 5 h (IQR = 2–12 h), while for ‘faux‐CM’ subjects, the median was 16 h (IQR = 5.5–66 h) (*P*‐value = 0.27). As previously reported, there was a strong correlation between retinal and brain vessel sequestration in the samples used in this study (Spearman rho = 0.77, *P*‐value = 0.01), supporting the direct relevance of processes in the retina to those occurring in the brain.^13^ When present, MMP8 staining was exclusively intravascular, while fibrinogen staining was seen both intravascularly (suggestive of microthrombus formation) and in the perivascular region (indicating vascular leak; Figure 1b). In subjects with ‘true‐CM’, MMP8 staining was present in a median of 88% (IQR = 74–91%) of examined capillaries, compared with 14% (IQR = 3.8–24%) of capillaries in subjects with ‘faux‐CM’ (*P*‐value = 0.001) (Figure 1c). Fibrinogen leak was detected in a median of 43% (IQR = 31–49%) of examined capillaries in subjects with ‘true‐CM’, compared to 4.6% (IQR = 1.9–9%) of capillaries in ‘faux‐CM’ subjects (*P*‐value = 0.03; Figure 1d). Co‐localisation of intravascular MMP8 and perivascular fibrinogen leak was found exclusively in capillaries with heavy sequestration of parasitised erythrocytes in subjects with ‘true‐CM’ and occurred in a median of 41% (IQR = 28–49%) of examined capillaries, compared to only 1.8% (IQR = 0–3.9%) of capillaries in subjects with ‘faux‐CM’ (*P*‐value = 0.01; Figure 1e). Fibrinogen leak was rarely seen in the absence of MMP8 staining in either group (≤ 5% of capillaries, Figure 1f), although this was significantly more common in subjects with ‘faux‐CM’ compared to ‘true‐CM’ (*P*‐value = 0.03).

**Table 1:** Characteristics and post‐mortem diagnosis of study subjects

Sample	Postmortem diagnosis	Age (months)	Sex	Time from admission to death (h)	Admission parasite density (parasites per µL)	Retinopathy	True‐ or Faux‐CM
1	CM	25	F	12	9718	+	True
2	CM	89	F	2	78 904	+	True
3	CM	144	M	8	308 720	+	True
4	CM	72	F	3	1729	+	True
5	CM	79	M	5	48 160	+	True
6	CM and severe anaemia	18	F	2	572 880	+	True
7	CM	42	F	24	364	+	True
8	Pneumonia, Reye's syndrome	35	M	22	159 434	−	Faux
9	Likely cause of death is anaemia	28	M	7	4139	−	Faux
10	Severe anaemia, hepatitis	8	F	1	788	−	Faux
11	Severe pneumonia	14	F	84	35 000	−	Faux
12	Severe pneumonia with spread to meninges	34	M	60	1 040 520	−	Faux
13	Fatal pneumonia	21	M	10	226 800	−	Faux

Post‐mortem examination of brain and retinal vasculature revealed no indication of cerebral malaria (CM) in six of the cases, who all had alternative non‐CM causes of death. The median age of the subjects was 34 months. M, male; F, female; +, malarial retinopathy present; −, malarial retinopathy not present.

## Discussion

Preceding work has indicated that increased expression of *MMP8* in blood leukocytes is associated with CM,^6^, ^7^ although systemic concentrations of MMP8 in blood did not differ significantly between severe and uncomplicated malaria.^5^, ^7^ This led us to hypothesise that localised release of MMP8 may be critical, perhaps occurring preferentially in tissues with *P. falciparum*‐infected erythrocyte sequestration. Indeed, we have previously shown that *P. falciparum* schizont extract effectively triggers MMP8 release from neutrophils.^7^ Here, we report extensive MMP8 release in the retinal microvasculature of subjects with ‘true‐CM’, which strongly co‐localises with fibrinogen leak from capillaries with prominent sequestration. Importantly, fibrinogen leak was rarely seen in the absence of MMP8 staining. Whilst these findings do not prove that MMP8 causes vascular leak, given what is known about the effects of MMP8, it seems highly plausible.

Activated neutrophils release large amounts of MMP8, so our findings suggest that neutrophil degranulation occurs in response to sequestered parasites. Despite neutrophils being the first immune cells to respond to infection, their role in malaria immunopathology is still not well understood.^14^ Neutrophils are typically increased in the peripheral blood of subjects with CM.^6^ While evidence of accumulation of intact neutrophils in cerebral and retinal vasculature is scarce, we have shown that other neutrophil products such as neutrophil extracellular traps do accumulate in the retinal microvasculature at sites of *P. falciparum‐*infected erythrocyte sequestration.^15^ We speculate that neutrophils in the retinal and cerebral microvasculature of patients with CM are intensely stimulated by the large numbers of sequestered parasites in these vessels and degranulate releasing MMP8, which is retained locally at high concentrations because of the obstruction of blood flow by the sequestered parasites. This could explain the apparent paradox that systemic levels of MMP8 are generally similar in severe and uncomplicated malaria.

Our findings support previous studies indicating that CM is associated with a compromised vascular endothelial barrier in the retina and the brain microvasculature, allowing vascular leakage.^2^, ^4^, ^16^ MMP8 can directly disrupt tight junctions through its proteolytic activity.^10^ In a prospective study of retinal angiography in children from the same setting, focal leakage of the blood–retina barrier was strongly associated with severe brain swelling on the MRI and death.^4^ This leakage developed rapidly over a few hours at foci within the superficial and deep retinal capillary plexi and resulted in profound egress of intravascular fluid and blood into the retina. Our data reported in this manuscript implicate MMP8 into this pathway to death. Although CM pathogenesis is complex and MMP8 is probably not the only factor responsible, it may be an important contributor and a tractable therapeutic target.

Limitations of this study include its small size, absence of paired plasma samples to measure systemic MMP8 concentrations and its observational nature, which precludes conclusions about causality. All samples in our study came from Malawian children, so future studies should examine other groups of subjects (including adults) from different settings to confirm the generalisability of these results.

Our study did not directly address the role of *P. falciparum* or the mechanisms of coma in the subjects classified as ‘faux‐CM’, and this remains an important open question. Autopsy‐defined causes of death in this group were heterogeneous, and coma may have been a consequence of late presentation to hospital, in a pre‐terminal stage of illness. Whether malaria could potentially contribute to coma in these subjects, through mechanisms independent of cerebral sequestration, is currently under investigation.

Overall, our work strengthens understanding of the role of MMP8 in CM pathogenesis and provides further evidence supporting a pathogenic role of neutrophils. Revealing factors that contribute to severe malaria pathogenesis should eventually lead to the development of adjunctive treatments targeted at acute focal neurovascular barrier breakdown that will reduce mortality.

## Methods

### Study design and subjects

We used archived post‐mortem ocular tissue samples from 13 Malawian children (Figure 1a), collected as part of a previously reported study.^13^, ^17^ These children met the World Health Organization clinical criteria for CM at initial clinical presentation: they were in coma and had peripheral asexual *P. falciparum* parasitaemia.^13^, ^17^ Retinal examination was undertaken in all subjects prior to death to determine the presence or absence of malarial retinopathy. Post‐mortem examination revealed that despite similar presentations, only 7 of the 13 children had sequestration of *P. falciparum*‐infected erythrocytes in the microvasculature of the brain (defining them as ‘true‐CM’ cases).^13^ The other 6 children lacked cerebral sequestration and were found at autopsy to have other causes of coma and death (‘faux‐CM’ controls, Table 1). ‘True‐CM’ includes histological classifications CM1 and CM2, while ‘faux‐CM’ refers to histological classification CM3.^2^, ^17^ Cases and controls were not individually matched. Sequestration of parasites in the retina and brain of the subjects used in this study had been quantified as previously described.^13^

### Immunofluorescent staining

Paraffin sections, 4 μm thick, were deparaffinised in xylene, rehydrated in ethanol and then treated with Uni‐TRIEVE (Universal Mild Temperature Retrieval Solution, Generon, Slough, UK) at 60°C. Normal horse serum (Vector Laboratories, Burlingame, CA, USA) was used to block non‐specific staining before samples were incubated with primary antibodies [anti‐MMP8 monoclonal rabbit antibody clone: EP1252Y (ab81286; Abcam, Cambridge, UK); anti‐fibrinogen goat polyclonal antibody (PA1‐26809; ThermoFisher, Waltham, MA, USA)] overnight at 4°C. Fluorescent secondary antibodies [donkey anti‐goat IgG H&L (Alexa Fluor® 647, ab150135, Abcam); goat anti‐rabbit IgG H&L (Alexa Fluor® 488, ab150081, Abcam)] were added for 30 min in the dark, and then, slides were rinsed and mounted with a 4′,6‐diamidino‐2‐phenylindole (DAPI) containing medium (VECTASHIELD Antifade Mounting Medium with DAPI, Vector Laboratories).

### Image analysis and scoring

An inverted Widefield Microscope with light‐emitting diode (LED) illumination (Axio Observer 7; Zeiss, Oberkochen, Germany) and a highly sensitive camera (Flash 4; Hamamatsu, Hertfordshire, UK) were used for imaging. Fluorescent images were processed using open source imaging software (Fiji). The images were scored for MMP8 and perivascular fibrinogen staining by two independent assessors (PN and SW), blinded to the CM status of the subjects to eliminate bias in scoring. Co‐localisation of MMP8 and fibrinogen was defined as the presence of perivascular fibrinogen staining around a capillary with intravascular MMP8 staining. From these 13 subjects, 169 images were collected and a mean of 34 capillaries per subject was scored. The difference in the number of vessels scored between the two groups was not significant, with ‘true‐CM’ capillaries IQR = 24–39 and SD = 8.4 and ‘faux‐CM’ capillaries IQR = 25–64 and SD = 27. To combine the scores from the two assessors, we used the mean score per subject for each assessed feature.

### Statistical analysis

Quantitative data were not transformed, and no data were excluded from analysis. GraphPad Prism 8 (GraphPad Software, San Diego, CA, USA) was used for all statistical analyses. The Mann–Whitney test was used to compare the scores between faux‐ and true‐CM groups. Spearman correlation was used to examine the relationship between brain and retinal vessel sequestration. All tests were two‐sided using a significance threshold of 5%.

### Ethical considerations

The core and specific studies all received approval from the research ethics committee at the University of Malawi College of Medicine (P. 11/07/593), Michigan State University and the Royal Liverpool and Broadgreen University Hospital Trust (n. 3690). Research was performed in accordance with the Declaration of Helsinki.

## Conflict of interest

The authors declare no conflict of interest.

## Author contributions

**Athina Georgiadou:** Conceptualization; Formal analysis; Funding acquisition; Investigation; Methodology; Supervision; Visualization; Writing‐original draft; Writing‐review & editing. **Praveena Naidu:** Formal analysis; Methodology; Writing‐review & editing. **Sophie Walsh:** Formal analysis; Writing‐review & editing. **Steve Kamiza:** Resources; Writing‐review & editing. **Valentina Barrera:** Resources; Writing‐review & editing. **Simon P Harding :** Resources; Writing‐review & editing. **Christopher A Moxon:** Conceptualization; Writing‐review & editing. **Aubrey J Cunnington:** Conceptualization; Supervision; Writing‐original draft; Writing‐review & editing.
